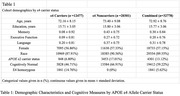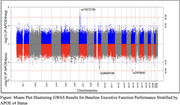# Cross‐Ancestry Meta‐Analysis of *APOE‐ε4* Stratified GWAS of Longitudinal Cognitive Decline in Older Adults

**DOI:** 10.1002/alz.090846

**Published:** 2025-01-03

**Authors:** Alex G. Contreras, Skylar Walters, Jaclyn M. Eissman, Derek B. Archer, Alexandra N. Regelson, Alaina Durant, Shubhabrata Mukherjee, Michael L. Lee, Seo‐Eun Choi, Phoebe Scollard, Emily H. Trittschuh, Jesse Mez, William S. Bush, Brian W. Kunkle, Adam C. Naj, Katherine A. Gifford, Murat Bilgel, Amanda B Kuzma, Michael L. Cuccaro, Carlos Cruchaga, Margaret A Pericak‐Vance, Lindsay A. Farrer, Li‐San Wang, Gerard D. Schellenberg, Richard Mayeux, Jonathan L. Haines, Angela L. Jefferson, Walter A. Kukull, C. Dirk Keene, Andrew J. Saykin, Paul M. Thompson, Eden R. Martin, Marilyn S. Albert, Sterling C. Johnson, Corinne D. Engelman, Luigi Ferrucci, David A. Bennett, Lisa L. Barnes, Julie A. Schneider, Susan M. Resnick, Reisa A Sperling, Paul K. Crane, Logan C. Dumitrescu, Timothy J. Hohman

**Affiliations:** ^1^ Vanderbilt Memory & Alzheimer’s Center, Vanderbilt University Medical Center, Nashville, TN USA; ^2^ Vanderbilt Genetics Institute, Vanderbilt University Medical Center, Nashville, TN USA; ^3^ Vanderbilt Memory and Alzheimer’s Center, Vanderbilt University Medical Center, Nashville, TN USA; ^4^ Vanderbilt Genetics Institute, Institute for Medicine and Public Health Vanderbilt University Medical Center, Nashville, TN USA; ^5^ University of Washington, School of Medicine, Seattle, WA USA; ^6^ University of Washington, Seattle, WA USA; ^7^ VA Puget Sound Health Care System, Seattle, WA USA; ^8^ Boston University Chobanian & Avedisian School of Medicine, Boston, MA USA; ^9^ Department of Population and Quantitative Health Sciences, Institute for Computational Biology, Case Western Reserve University, Cleveland, OH USA; ^10^ John P. Hussman Institute for Human Genomics, University of Miami Miller School of Medicine, Miami, FL USA; ^11^ University of Pennsylvania Perelman School of Medicine, Philadelphia, PA USA; ^12^ National Institute on Aging, National Institutes of Health, Baltimore, MD USA; ^13^ NeuroGenomics and Informatics Center, Washington University School of Medicine, St Louis, MO USA; ^14^ 1501 NW 10th Avenue, Miami, FL USA; ^15^ Boston University School of Public Health, Boston, MA USA; ^16^ The Institute for Genomic Medicine, Columbia University Medical Center, New York, NY USA; ^17^ Taub Institute for Research on Alzheimer’s Disease and the Aging Brain, Columbia University Medical Center, New York, NY USA; ^18^ Department of Laboratory Medicine and Pathology, University of Washington, Seattle, WA USA; ^19^ Indiana Alzheimer’s Disease Research Center, Indianapolis, IN USA; ^20^ University of Southern California, Los Angeles, CA USA; ^21^ Johns Hopkins University, Baltimore, MD USA; ^22^ Alzheimer’s Disease Research Center, University of Wisconsin School of Medicine and Public Health, Madison, WI USA; ^23^ Translational Gerontology Branch, National Institute on Aging, NIH, Baltimore, MD USA; ^24^ Rush University, Chicago, IL USA; ^25^ Rush University Medical Center, Chicago, IL USA; ^26^ Rush Alzheimer’s Disease Center, Rush University Medical Center, Chicago, IL USA; ^27^ Harvard Medical School, Cambridge, MA USA

## Abstract

**Background:**

The Apolipoprotein E ε4 (*APOE*‐ε4) allele is common in the population, but acts as the strongest genetic risk factor for late‐onset Alzheimer’s disease (AD). Despite the strength of the association, there is notable heterogeneity in the population including a strong modifying effect of genetic ancestry, with the *APOE‐*ε4 allele showing a stronger association among individuals of European ancestry (EUR) compared to individuals of African ancestry (AFR). Given this heterogeneity, we sought to identify genetic modifiers of *APOE‐*ε4 related to cognitive decline leveraging *APOE‐*ε4 stratified and interaction genome‐wide association analyses (GWAS).

**Method:**

We leveraged a comprehensive harmonized cognitive dataset from nine well‐characterized aging and AD cohorts, representing African and European ancestries (total of 32,751 individuals, including 10,969 EUR ε4 carriers, 18,385 EUR non‐carriers, 1,495 AFR ε4 carriers, and 1,902 AFR non‐carriers). We then conducted *APOE* stratified and interaction GWAS and post GWAS analyses on longitudinal composite measures of memory, executive function, and language. To capture longitudinal cognitive trajectories, linear mixed effects models were employed, focusing on the rate of cognitive decline over time. Post GWAS analysis included eQTL analysis, gene‐set/pathway‐set analysis, and an assessment of genetic correlations.

**Result:**

We identified novel loci for baseline executive function performance including two that are specific to *APOE*‐ε4 carriers (rs28609108 on chromosome 9 and rs2959641 on chromosome 15) and one that is specific to non‐carriers (rs73072750 on chromosome 7). The effect sizes of these three loci remained consistent when subset by ancestry, achieving statistical significance in EUR, and nominal significance in AFR. Post GWAS eQTL analyses implicate the glutamate receptor subunit gene (*GRIN3A*) among *APOE*‐ε4 carriers, while the integrin gene (*ITGB8)*. Additionally, both *GRIN3A* and *ITGB8* have been previously implicated in AD/cognition GWAS, underscoring their relevance in the context of *APOE*‐ε4.

**Conclusion:**

Our study has shed light on several novel genetic factors that relate to late‐life cognitive performance in an *APOE*‐specific manner. These findings enrich our understanding of the genetic landscape influencing cognitive decline. Further research is imperative to unravel how these genetic variations affect biological processes and the potential in guiding therapeutic strategies.